# The Recent Use, Patient Satisfaction, and Advancement in Digital Smile Designing: A Systematic Review

**DOI:** 10.7759/cureus.62459

**Published:** 2024-06-16

**Authors:** Amulya Jain, Purnendu Bhushan, Mousumi Mahato, Bhavini B Solanki, Debanwita Dutta, Sadananda Hota, Anjana Raut, Arun K Mohanty

**Affiliations:** 1 Prosthodontics, Kalinga Institute of Dental Sciences, Kalinga Institute of Industrial Technology (KIIT) DU, Bhubaneswar, IND; 2 Prosthodontics, Kalinga Institute of Medical Sciences, Kalinga Institute of Industrial Technology (KIIT) DU, Bhubaneswar, IND; 3 Prosthodontics and Crown and Bridge, Kalinga Institute of Dental Sciences, Kalinga Institute of Industrial Technology (KIIT) DU, Bhubaneswar, IND

**Keywords:** aesthetics dentistry, digital dentistry, smile design, smile, digital smile design

## Abstract

Digital smile designing (DSD) is a concept of dentistry which combines the old and the new and becomes a different world in the world of smile aesthetics and functionality. Dental aesthetics is not just a cosmetic issue but a multidimensional part of oral health that has a great impact on psychological well-being, social life, functional capabilities, and, hence, the quality of life. To put it simply, the recognition of its significance stresses the necessity of complete dental care which is the one that combines beauty and function as well as health. This systematic review aims to analyze the recent use and patient satisfaction of DSD and to show the recent advances in DSD. A thorough literature search was conducted across the online databases for articles about the implementation of digital smile analysis in dentistry. The articles that were published between 2013 and 2023 on DSD were selected which included randomized and non-randomized trials and observational studies covering the effectiveness, advantages, and patients' opinions about the treatment. The National Institutes of Health tool was applied for bias assessment. Ten studies were selected to address the use of DSD in dentistry based on the inclusion criteria. The findings from these studies suggest that DSD is useful in improving communication, reducing working time, minimizing errors, enhancing patient satisfaction, and providing clinical adequacy for final prosthetic pieces, indicating the usefulness of this approach in dental procedures. Smile designing using digital technologies has the potential to improve dental aesthetics and treatment procedures while showcasing their reliability and clinical effectiveness.

## Introduction and background

Dental aesthetics is a critical aspect within the field of contemporary dentistry, extending its impact beyond the mere visual enhancement of a smile. It encompasses a complex interplay of factors that influence not only cosmetic appearance but also psychological well-being, social interactions, functional efficiency, and overall quality of life. The acknowledgment of its significance emphasizes the necessity for integrative dental care that seamlessly combines aesthetic considerations with functional and health-related outcomes [1].

Psychological well-being is impacted by self-esteem and confidence. Those who are satisfied with their dental aesthetics often exhibit higher self-esteem levels, allowing them to engage more confidently in social interactions and professional settings [2]. The smile is often the first facial feature people notice. A harmonious and attractive smile can leave a lasting positive impression, whether in personal relationships or professional encounters.

The position of teeth affects functionality because it determines efficiency during chewing which in turn promotes good digestion leading to better overall physical condition; additionally, aligned teeth also reduce chances of getting tooth-related problems like cracks or sensitivity [1,2]. A radiant smile has been linked with attractiveness, success, and a sound body.

Smile designing digitally utilizes up-to-date technological advancements. These include CAD/CAM systems, 3D imaging, and virtual simulations, among other tools. Planning becomes accurate because this enables exactness during customization as well as visualization of dental treatments from different perspectives [1]. In addition, it reduces errors while saving time on the chairside, thus enhancing the predictability of treatment outcomes [2].

Dentists can tailor treatments based on individual facial features, preferences, and functional needs. This patient-centric approach enhances satisfaction and ensures treatments align with patients' expectations. The integration of digital technologies allows for a harmonious blend of aesthetics and functionality. Dentists can simulate the final outcome, ensuring the smile not only looks appealing but also functions optimally, addressing issues like bite alignment, lip support, and speech clarity [3,4].

As digital smile designing (DSD) technologies rapidly evolve, there is a crucial need to understand how these advancements are being integrated into clinical practice leading to the best clinical outcomes [5]. Understanding patient satisfaction with DSD techniques is essential for ensuring that these treatments meet patient expectations and contribute to their overall well-being. This study will provide insights for optimizing treatment protocols and achieving superior aesthetic and functional results with DSD.

The main reason for focusing on recent use and developments in DSD is its ability to transform dentistry forever. DSD offers a blend of artistry, precision, and innovation, promising enhanced outcomes, improved patient experiences, and a brighter future for aesthetic dentistry [6]. The purpose of this study is to evaluate the recent use, advancement, and patient satisfaction in DSD techniques.

## Review

Methodology

**Figure 1:**
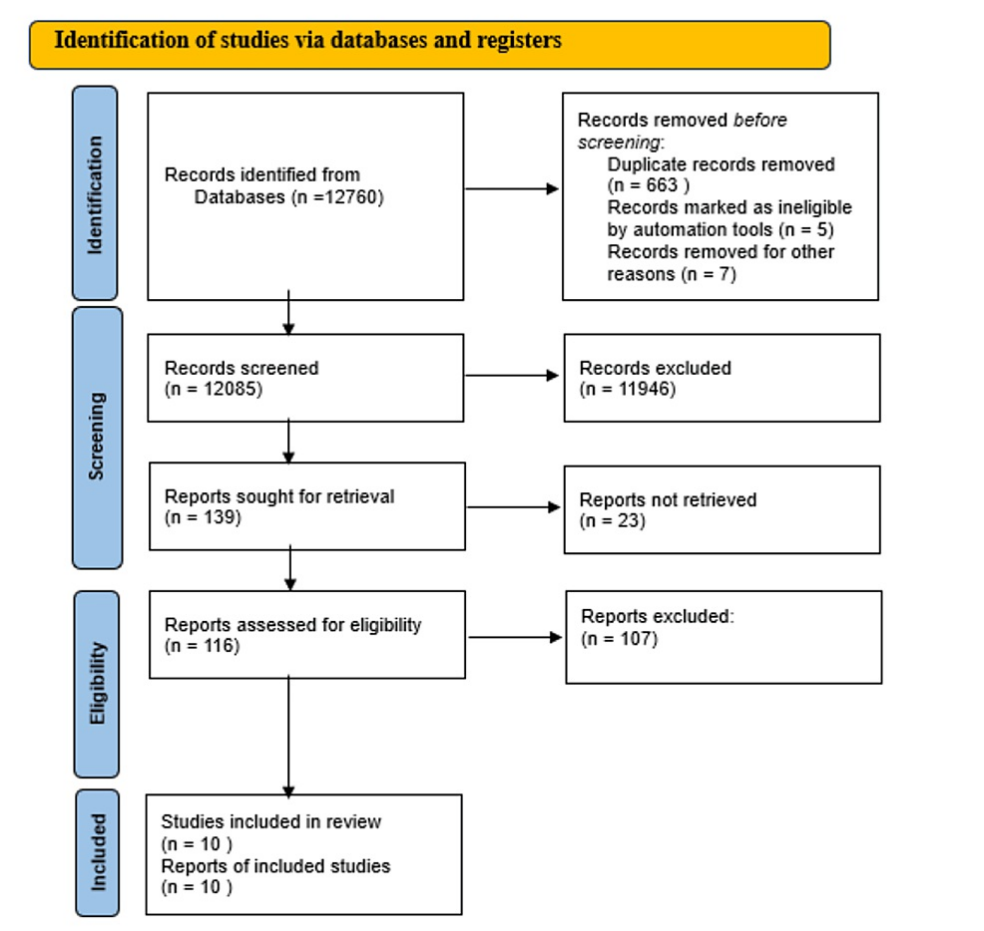
PRISMA 2020 guidelines for new systematic reviews which included searches of databases and registers only PRISMA: Preferred Reporting Items for Systematic Reviews and Meta-Analyses

This systematic review was conducted according to the Preferred Reporting Items for Systematic Reviews and Meta-Analyses (PRISMA) guideline [7] (Figure 1) and has been registered on the International Prospective Register of Systematic Reviews (PROSPERO) (ID: CRD42023457194). The Population, Intervention, Comparison, and Outcome (PICO) question was designed to identify and structure the fundamental components of the systematic review. How useful is DSD and how does it affect patient satisfaction? What are the advances in DSD? The PICO research model was used to implement this research question. The population assessed was adult patients who require aesthetic dental treatment with the intervention of DSD approaches. No comparison parameter was defined for this systematic review. The outcomes assessed are that DSD is more useful, patients' satisfaction is higher, and there are advancements in DSD.

The population involved consisted of adult patients who needed aesthetic dental treatment, with the help of DSD approaches. No comparison parameter was used. The outcomes assessed are that DSD is more useful, patients' satisfaction is higher, and there are advancements in DSD. The articles that were published between 2013 and 2023 on DSD were selected which included randomized and non-randomized trials and observational studies covering the effectiveness, advantages, and patients' opinions about the treatment. Articles published in the English language were included. In the exclusion criteria, the patients with diseases such as specific immunological disorders and craniofacial anomalies were excluded as well as researches with conflict of interests; "in vitro" studies, abstracts, and case reports; and reviews and reports on other technologies.

Search Strategy

A systematic search was conducted for this review in the following electronic databases: PubMed, Scopus, Web of Science, ScienceDirect, and Cochrane Library. The searches were conducted from 2013 to 2023. Electronic search strategies were customized according to each database. The search strategy was restricted to the English language. The Medical Subject Headings (MeSH) terms and related keywords like "aesthetic dentistry," "digital smile design," "smile aesthetics," etc. were paired with Boolean operators 'AND' and 'OR' for building keywords to search across different databases. The PubMed search was held by the following approach: ((aesthetic dentistry) [All Fields] AND (((digital dentistry) [MeSH Terms] OR digital smile design OR DSD) [All Fields] OR dental photography)) [MeSH Terms] AND ((((smile aesthetics)[MeSH Terms] OR ´smile`AND digital dentistry) [All Fields] OR (digital smile analysis) "[MeSH Terms].

Study Selection

Two independent reviewers conducted a multi-stage selection process. The first stage was to review the titles to eliminate irrelevant studies. The second stage involved reviewing and filtering the summaries based on the type of study, type of digital intervention, and number of patients and outcome variables. The third stage involved fully reading each text and extracting the data using a predetermined data extraction form to confirm the eligibility of the studies.

Data Extraction

The data collected from different articles was analyzed by author names, publication year, study design, male-to-female ratio, and mean and age range in years. The data was collected by reviewers independently and converted in the form of a data extraction sheet, and the individual study results were compiled.

Study Risk of Bias and Quality Assessment

The quality of the included studies was assessed independently by two independent reviewers to evaluate the methodological quality and risk of bias of all included articles. After removing duplicate sources, study titles and abstracts were reviewed to ensure they were relevant. Following that, studies that fulfilled the inclusion criteria were included after a full-text review. Critical appraisal of randomized controlled trials (RCTs) and observational studies was done according to the National Institutes of Health (NIH) tool [8]. The quality assessment of RCTs and observational studies was done by the questionnaire as per the NIH tool. "High risk of bias" translates to a rating of "poor quality." In contrast, "low risk of bias" translates to a rating of "good quality."

Results

The search initially produced 12760 articles. The titles and abstracts were scrutinized, and the irrelevant and duplicate articles were eliminated leading to 139 full-text articles which were then evaluated for eligibility. The inclusion/exclusion criteria mentioned above were used to select a total of 10 articles. Figure 1 describes the study selection process for this systematic review as per the PRISMA protocol. Nine observational studies and 1 RCT were selected. During the data extraction process, two independent authors evaluated the risk of bias. The included studies examined 344 subjects, and it should be noted that the majority of them were between 18 and 50 years old. The evaluation was performed on various DSD software. The results of the individual studies were tabulated (Table 1).

**Table 1:** Results of all the individual studies included in this systematic review RCT: randomized controlled trial; DSS: digital smile system; DSD: digital smile design; DSP: digital smile planning; RED: recurring esthetic dental; VAS: Visual Analogue Scale; ANOVA: analysis of variance; PMMA: poly(methyl methacrylate); PLVs: porcelain laminate veneers; DP: dental proportion; STL: Standard Tessellation Language

Authors and year of publication	Study design	Software equipment	Sample		Parameter assessed	Assessment tool	Main outcome
Vivek et al. (2013) [9]	Observational study	A Vita classical shade guide (VITA Zahnfabrik, Bad Säckingen, Baden-Württemberg, Germany) was used for shade matching. Digital color matching software was used for the digitally assisted shade matching method	20 subjects		Compares conventional shade matching method with digitally assisted shade matching method	Chi-squared analysis	A new computer matching method was proposed and found to be superior to the conventional shade matching method
Cattoni et al. (2016) [10]	Observational study two-year follow-up	2D DSS (Digital Smile System Srl, Italy); 3D DSS (EGS Srl, Italy)	28 patients (9 males and 19 females)	19-53 years	3D DSP technique used in the previsualization stage prior to milling (PMMA) mock-ups in the process of creating PLVs using a CAD/CAM system	VAS	The 3D digital planning technique is a predictable and minimally invasive technique, allows easy diagnosis, and improves communication with the patient
Liberato et al. (2019) [11]	RCT	VITA Classical A1-D4 and VITA Toothguide 3D-MASTER with 29 tabs (VITA Zahnfabrik) with and without the aid of a light-correcting device (Smile Lite; Smile Line, St-Imier, Switzerland) (TRIOS; 3Shape, Copenhagen, Denmark) and a spectrophotometer (VITA Easyshade Advance 4.0; VITA Zahnfabrik)	28 patients		Compares the reliability of different visual and instrumental methods for dental shade matching	Fleiss' kappa statistical test	Instrumental methods for color shade matching were more reliable than the visual methods tested
Lavorgna et al. (2019) [12]	Observational study	Emerald, TRIOS, photogrammetry, and DSS	12 patients	25-35 years of age	Differences in measurements of the different tool data (Emerald, TRIOS, photogrammetry, and DSS) in terms of reliability and accuracy	Statistical analysis was performed using statistical software	No significant differences emerged in the measurements made with the different scanners
Lo Giudice et al. (2020) [13]	Observational study	2D DSS (version 1.11.1-alpha.1, Digital Smile System Srl); DSS CAD software (DSS3D. Beta.12977, EGS Srl)	10 patients		Matching percentage of prototyped and milled mock-ups with 3D project. Comparison of linear measurements (mm^3^) performed on 3D project, prototyped, and prototyped scanned anterior mock-ups	Paired Student's t-test. Two-way ANOVA was used	Both prototype and milled mock-ups showed a slight dimensional increment in comparison with the original 3D project. Prototyped mock-ups demonstrated few dimensional changes from the original 3D project compared to the milled mock-ups as well as a greater clinical adaptation
Ortensi et al. (2022) [14]	Observational study	2D DSS (Digital Smile System Srl); 3D Exocad® Dental CAD 2.2 Valletta software (Exocad GmbH, Darmstadt, Hessen, Germany); 3Shape 3D Viewer® software (3Shape)	18 women and 12 men	20-50 years of age,	Evaluates the production of customized composite veneers starting from a 2D digital preview using the DSS	The Friedman, Bonferroni, and Dunn post hoc tests were used	The final composite customized veneers were comparable with the 2D and 3D plans, confirming the clinical adequacy of the final prosthetic pieces
Cattoni et al. (2021) [15]	RCT four-year follow-up	Smile Lynx CAD software (88Dent, Pero, Lombardy, Italy)	50 patients	46 and 85 years	Digital smile designed computer-aided. Immediate loading of the implants	Verbal Rating Scale	All patients treated with a digital method reported lower values of during-surgery and post-surgery pain compared to patients rehabilitated using traditional treatment
Varghese et al. (2021) [16]	Observational study	Adobe Photoshop CS5 (Adobe Systems, Inc., San Jose, California, United States)	150 subjects (male-to-female ratio: N/A)	18-25 years of age	Golden ratio, golden rectangle, and RED proportion were assessed	Paired Student's t-test (level of significance: p≤0.05)	The golden ratio appears to be relevant to link the consecutive widths of the maxillary anterior teeth. The golden rectangle concept is useful for selecting aesthetically attractive dimensions for maxillary central incisors. The RED proportion is an inadequate approach for relating the sequential widths of the maxillary anterior teeth, according to DP and analyses
Lv et al. (2022) [17]	Observational study	3D treatment simulation as well as 2D DSD	11 patients	19-36 years of age	Difference between intuitiveness, understanding, and satisfaction in 3D treatment stimulation or 2D DSD plus wax-up technique	VAS	3D treatment simulation showed obvious advantages in the aspects of intuitiveness (9.7±0.5 vs 6.4±1.4) and treatment understanding (9.1±0.8 vs 6.6±1.5), and the satisfaction rates were also higher (9.0±0.6 vs 7.1±1.8)
Chisnoiu et al. (2023) [18]	Observational study	3Shape scanner and STL images as well as initial smile evaluation photos were analyzed, superposed, and processed using the 3Shape Dental System Software (3Shape)	5 patients	20-25 years of age	Compares the aesthetic aspects and assessment of conventional versus digital prefigurative methods	The aesthetic assessment questionnaire was created by the authors based on the research of Mocelin et al.	The analysis has shown a balanced assessment of the aesthetic criteria without any significant difference between the analog and digital prefigurative methods

Bias Assessment Within Individual Studies

To assess the risk of bias in the included RCT and observational studies, the NIH tool [8] was used (Table 2 and Table 3). The included RCT was considered an unclear risk of bias translating to fair quality. For observational studies, the risk of bias was considered low risk translating to good quality in three studies and unclear risk translating to fair quality in six studies. Each study design, case selection, and DSD approach had a different degree of heterogeneity among the studies.

**Table 2:** Quality assessment of randomized trials according to the NIH tool RCT: randomized controlled trial; NIH: National Institutes of Health; NR: not related

	Cattoni et al. 2021 [15]
1. Was the study described as randomized, a randomized trial, a randomized clinical trial, or an RCT?	Yes
2. Was the method of randomization adequate (i.e., use of randomly generated assignment)?	Yes
3. Was the treatment allocation concealed (so that assignments could not be predicted)?	Yes
4. Were study participants and providers blinded to treatment group assignment?	NR
5. Were the people assessing the outcomes blinded to the participants' group assignments?	NR
6. Were the groups similar at baseline on important characteristics that could affect outcomes (e.g., demographics, risk factors, comorbid conditions)?	NR
7. Was the overall drop-out rate from the study at endpoint 20% or lower of the number allocated to treatment?	NR
8. Was the differential drop-out rate (between treatment groups) at endpoint 15 percentage points or lower?	NR
9. Was there high adherence to the intervention protocols for each treatment group?	Yes
10. Were other interventions avoided or similar in the groups (e.g., similar background treatments)?	Yes
11. Were outcomes assessed using valid and reliable measures and implemented consistently across all study participants?	Yes
12. Did the authors report that the sample size was sufficiently large to be able to detect a difference in the main outcome between groups with at least 80% power?	NR
13. Were outcomes reported or subgroups analyzed prespecified (i.e., identified before analyses were conducted)?	NR
14. Were all randomized participants analyzed in the group to which they were originally assigned, i.e., did they use an intention-to-treat analysis?	Yes
Quality rating (good, fair, or poor)	Fair

**Table 3:** Quality assessment of non-randomized trials according to the NIH tool NIH: National Institutes of Health; NR: not related

	Vivek et al. (2013) [9]	Cattoni et al. (2016) [10]	Liberato et al. (2018) [11]	Lavorgna et al. (2019) [12]	Lo Giudice et al. (2020) [13]	Ortensi et al. (2021) [14]	Varghese et al. (2021) [16]	Lv et al. (2022) [17]	Chisnoiu et al. (2023) [18]
1. Was the research question or objective in this paper clearly stated?	Yes	No	Yes	Yes	Yes	Yes	Yes	Yes	Yes
2. Was the study population clearly specified and defined?	Yes	Yes	Yes	Yes	Yes	Yes	Yes	Yes	Yes
3. Was the participation rate of eligible persons at least 50%?	NR	NR	Yes	Yes	NR	NR	NR	Yes	NR
4. Were all the subjects selected or recruited from the same or similar populations (including the same time period)? Were inclusion and exclusion criteria for being in the study prespecified and applied uniformly to all participants?	Yes	Yes	Yes	Yes	Yes	Yes	Yes	Yes	NR
5. Was a sample size justification, power description, or variance and effect estimates provided?	NR	NR	Yes	NR	No	NR	NR	Yes	NR
6. For the analyses in this paper, were the exposure(s) of interest measured prior to the outcome(s) being measured?	Yes	Yes	Yes	Yes	Yes	Yes	Yes	Yes	Yes
7. Was the timeframe sufficient so that one could reasonably expect to see an association between exposure and outcome if it existed?	NR	Yes	Yes	Yes	Yes	Yes	NR	Yes	Yes
8. For exposures that can vary in amount or level, did the study examine different levels of the exposure as related to the outcome (e.g., categories of exposure or exposure measured as continuous variable)?	Yes	Yes	Yes	Yes	Yes	Yes	No	Yes	No
9. Were the exposure measures (independent variables) clearly defined, valid, reliable, and implemented consistently across all study participants?	Yes	NR	Yes	Yes	Yes	Yes	Yes	Yes	Yes
10. Was the exposure(s) assessed more than once over time?	NR	Yes	Yes	No	Yes	No	No	No	Yes
11. Were the outcome measures (dependent variables) clearly defined, valid, reliable, and implemented consistently across all study participants?	Yes	NR	Yes	Yes	Yes	Yes	NR	Yes	Yes
12. Were the outcome assessors blinded to the exposure status of participants?	NR	NR	NR	NR	NR	NR	NR	No	Yes
13. Was loss to follow-up after baseline 20% or less?	NR	Yes	NR	NR	NR	NR	NR	No	NR
14. Were key potential confounding variables measured and adjusted statistically for their impact on the relationship between exposure(s) and outcome(s)?	NR	NR	Yes	NR	NR	NR	NR	No	Yes
Quality rating (good, fair, or poor)	Fair	Fair	Good	Fair	Fair	Fair	Fair	Good	Good

*Results of Individual Studies* 

In 2016, Cattoni et al. [10] conducted a study using images and scanned files to visualize teeth and enhance smile design. Their subsequent research [15] in 2021 utilized an approach for planning fixture placements and crafting prostheses through CAD/CAM techniques. With the help of the research of Lavorgna et al. [12], the acceptability of the 2D method clearly went up for the person in the real scenario, and Lv et al. [17] demonstrated the fact that when patients use the treatment plans created by 3D digital simulation, the satisfaction rate is higher than the traditional one.

Moreover, the research of Chisnoiu et al. [18] and Lo Giudice et al. [13] changed the old logic of using only one technique as the analog traditional mock-up and lightweight digital mock-up have their own different advantages, which can help you make new ideas. Varghese et al.'s study [16] explored digital photo design and how it aided in the upliftment of the aesthetic appearance of the anterior maxillary teeth compared to Liberato et al. [11] who argued how a hybrid technique between the instrumental techniques and spectrum photometry technology ensures reliability and precision of shade matching in a clinical environment. Ortensi et al. [14] discovered that the heights determined from the photos and those assessed from the digital scans did not have any significant changes revealing that the discrepancies in mesial-distal widths mentioned in photographs and digital scans were connected to the transition from a 2D to a 3D environment. The study result conducted by Vivek et al. [9] showed how the digital technique was way better than the old methods in color identification possibility, the prospects of digital technology being able to increase the precision of color identification and the quality of the outcomes of the treatment.

Discussion

This systematic review discusses the importance of dental aesthetics, the rise of DSD, and reasons for focusing on recent advancements in the field. It emphasizes the psychological, social, functional, and economic significance of dental aesthetics, highlighting its role in self-esteem, first impressions, chewing improvement, and overall health indicators. The rationale for focusing on recent use and advancements in DSD is rooted in technological integration, precision, personalized patient experience, interdisciplinary collaboration, efficiency, and continuous innovation.

Our article reviews different studies that utilize different methodologies, including RCTs, observational studies, and quality assessments according to the NIH tool. They compare visual and instrumental methods for dental shade matching, aesthetic aspects, and assessment of conventional versus digital planning, digital smile planning techniques, DSD, and treatment simulations. The quality assessments evaluate the research questions, study populations, sample sizes, exposure and outcome measures, blinding, loss to follow-up, and potential confounding variables.

DSD is useful in several studies. For example, Cattoni et al. [10] in 2016 conducted a two-year follow-up observational study and found that previsualization was enhanced by the use of a 3D digital smile planning technique (3D-DSP) which was a predictable and minimally invasive technique that improved communication with the patient, reduced working time, and minimized errors associated with the classical prosthodontic manual step. Moreover, Ortensi et al. [14] in 2021, in an observational study, found that final composite customized veneers assessed by a 2D digital preview using the DSD were comparable with the 2D and 3D plans which confirmed the clinical effectiveness of the final prosthetic components. Additionally, Lv et al. [17] in 2022 reported in an observational study that, when compared, 3D treatment simulations were clearly superior in terms of intuitiveness, treatment understanding, and satisfaction rates to 2D DSD plus wax-up technique, as assessed by patients and dental specialists using the Visual Analogue Scale (VAS) for rating. Lavorgna et al. [12] further emphasized that to measure 2D and 3D identically, 2D images must be taken with the camera sensor parallel to the observed item and the surface of the observed subject must be flawlessly flat.

Liberato et al. [11] in 2018 supported the recommendation for the additional use of shade matching instruments alongside visual shade guides which improves the accuracy and consistency of color selection for dental restorations. Similarly, Vivek et al. [9] in 2013 demonstrated the potential of digital methods to improve communication between dentists and technicians, allowing for better color measurement and improved clinical outcomes. These studies concluded that the digitally assisted shade matching method was superior to the conventional method.

The study by Cattoni and others [10] in 2016 mentions that using 3D tech for planning smiles is easy, is less invasive, makes talking to patients simpler, cuts down on work time, and reduces mistakes. Also, in 2022, Lv and colleagues [17] discovered that 3D ways of showing treatment plans were better at making things clear and easy to understand and made patients happier compared to the older 2D methods plus wax models. So, these studies point out that, in general, patients liked the new digital and 3D methods more for their dental work.

Several software systems have been used for dental applications, each with its advances and benefits. The software systems include the 3Shape Dental System Software (Copenhagen, Denmark) which is used for processing Standard Tessellation Language (STL) images and initial smile evaluation photos in an observational study by Chisnoiu et al. [18] in 2023. This software enables the analysis, superposition, and processing of dental images and photos, contributing to the assessment of aesthetics and comparison of conventional versus digital methods. The 3Shape 3D Viewer software was also employed by Ortensi et al. [14] in a study they conducted in 2021 to evaluate the production of customized composite veneers. This software aids in facilitating the visualization and analysis of 3D dental plans, aiding in the comparison of linear measurements and final veneers to confirm the clinical adequacy of prosthetic pieces. Varghese et al. [16] in 2021 used Adobe Photoshop CS5 (Adobe Systems, Inc., San Jose, California, United States) and accomplished that the golden percentage theory and the golden rectangle concept are employed to determine visually appealing proportions for maxillary central incisors. Another software used was Smile Lynx CAD software (88Dent, Pero, Lombardy, Italy) which was utilized in the research conducted by Cattoni et al. [15] in 2021 to design computer-aided immediate loading of implants. Notably, advancement in CAD contributes to lower pain levels reported during and after surgery compared to traditional treatment. The Exocad Dental CAD 2.2 Valletta software (Exocad GmbH, Darmstadt, Hessen, Germany) is another software that was used in a study by Ortensi et al. [14] in 2021 to assess how well-customized composite veneers are produced. The benefit of this software is that it provides advanced capabilities for dental CAD design, aiding in the creation of customized composite veneers based on 2D digital previews.

Digital Smile System (DSS) (Italy) was also used for creating DSD and employed in multiple studies, including one by Cattoni et al. [10] in 2016. The advanced feature of this software was the previsualization stage before creating prosthetic pieces, contributing to minimally invasive techniques, improved diagnosis, and effective communication with patients. These advancements in software equipment have played a significant role in enhancing various aspects of dental procedures, including aesthetic assessment, prosthetic design, and treatment planning.

Lo Giudice et al. [13] in 2020 concluded in harmony with Cattoni et al. [10] that prototyped mock-ups showed smaller dimensional changes compared to milled mock-ups. Using a CAD/CAM system, 3D-DSP prosthetic dental restorations, particularly poly(methyl methacrylate) (PMMA) mock-ups, were created. This technology has been shown to improve communication with patients by allowing for a visual previsualization stage, which aids in conveying the planned outcome of the procedure. By utilizing 3D-DSP, the working time is reduced, and the errors that occur with traditional manual prosthodontic steps are reduced.

Several studies have demonstrated the efficacy of the 3D digital planning technique in improving patient communication, reducing working time, and minimizing errors in prosthodontic procedures. The digital smile planning technique has also been associated with a lower incidence during- and post-surgery pain compared to conventional treatment methods, enhancing patient experience and outcomes.

The results of quality assessment of non-randomized trials according to the NIH tool indicated varying levels of quality among the studies. Some studies clearly stated their research question/objective specified the study population and applied uniform inclusion/exclusion criteria. However, not all studies provided a sample size justification or power description. Most studies measured exposure before outcome and had a sufficient timeframe for associations to be seen. Overall, the quality assessment of non-randomized trials shows that while some studies demonstrated high quality in terms of study design and methodology, others had some deficiencies in reporting key aspects of the research.

The quality assessment of randomized trials according to NIH, as per the provided document, consisted of evaluating various aspects such as randomization adequacy, treatment allocation concealment, blinding, adherence to intervention protocols, avoidance of other interventions, and use of valid and reliable outcome measures. The overall quality rating was considered "fair."

Therefore, the findings from these studies suggest that DSD is useful in improving communication, reducing working time, minimizing errors, enhancing patient satisfaction, and providing clinical adequacy for final prosthetic pieces, indicating the usefulness of this approach in dental procedures. Some limitations can be identified based on the study design and data analysis methods which include small sample sizes which may limit the generalizability of the findings to the broader population. Also, the duration of follow-up in the studies varied, but observational studies generally included a relatively short period of follow-up. The inclusion criteria specified articles published in English or other languages for which an English translation was possible limiting the comprehensiveness of the review.

## Conclusions

The findings of our study are that digital techniques and 3D technologies have some benefits which include trueness in instrumental alignments, predictable methods of smile planning that are minimally invasive, and clinical adequacy of achieved prosthetic parts. Furthermore, the research in this area emphasizes the significance of improved patient satisfaction by the digital simulated design feature as a part of treatment. These findings together bind the potent impact of digital gadgets in enhancing cosmetic features and treatment procedures, which further approve their valuable capabilities and clinical efficiency. The studies covered in this article provide valuable insights into various aspects of dental shade matching, DSD, and aesthetic assessment methods and also demonstrate the potential benefits of instrumental methods for color shade matching over visual methods, the predictability and minimally invasive nature of 3D digital smile planning techniques, and the clinical adequacy of final prosthetic pieces produced through digital systems. Moreover, the studies underline the importance of digital simulated design in enhancing patients' satisfaction with treatment outcomes. These conclusions collectively highlight the potential of digital technologies in improving dental aesthetics and treatment procedures while showcasing their reliability and clinical effectiveness.
